# Critical shoulder angle combined with age predict five shoulder pathologies: a retrospective analysis of 1000 cases

**DOI:** 10.1186/s12891-017-1559-4

**Published:** 2017-06-15

**Authors:** Philipp R. Heuberer, Fabian Plachel, Lukas Willinger, Philipp Moroder, Brenda Laky, Leo Pauzenberger, Fritz Lomoschitz, Werner Anderl

**Affiliations:** 1 St. Vincent Shoulder and Sports Clinic Vienna, Baumgasse 20A, 1030 Vienna, Austria; 2 Department of Traumatology and Sports Injuries of the Paracelsus Medical University Salzburg, Salzburg, Austria; 3 Department of Orthopaedic Sports Medicine, Klinikum Rechts der Isar, Technical University Munich, Munich, Germany; 4 Department of Radiology at the St. Vincent Hospital Vienna, Vienna, Austria; 5 Austrian Research group for Regenerative and Orthopedic Medicine (AURROM), Vienna, Austria

**Keywords:** Critical shoulder angle, Cuff tear arthropathy, Glenohumeral osteoarthritis, Rotator cuff tear, Impingement

## Abstract

**Background:**

Acromial morphology has previously been defined as a risk factor for some shoulder pathologies. Yet, study results are inconclusive and not all major shoulder diseases have been sufficiently investigated. Thus, the aim of the present study was to analyze predictive value of three radiological parameters including the critical shoulder angle, acromion index, and lateral acromion angle in relationship to symptomatic patients with either cuff tear arthropathy, glenohumeral osteoarthritis, rotator cuff tear, impingement, and tendinitis calcarea.

**Methods:**

A total of 1000 patients’ standardized true-anteroposterior radiographs were retrospectively assessed. Receiver-operating curve analyses and multinomial logistic regression were used to examine the association between shoulder pathologies and acromion morphology. The prediction model was derived from a development cohort and applied to a validation cohort. Prediction model’s performance was statistically evaluated.

**Results:**

The majority of radiological measurements were significantly different between shoulder pathologies, but the critical shoulder angle was an overall better parameter to predict and distinguish between the different pathologies than the acromion index or lateral acromion angle. Typical critical shoulder angle-age patterns for the different shoulder pathologies could be detected. Patients diagnosed with rotator cuff tears had the highest, whereas patients with osteoarthritis had the lowest critical shoulder angle. The youngest patients were in the tendinitis calcarea and the oldest in the cuff tear arthropathy group.

**Conclusions:**

The present study showed that critical shoulder angle and age, two easily assessable variables, adequately predict different shoulder pathologies in patients with shoulder complaints.

## Background

Acromion morphology has been related to shoulder pathologies, thus various attempts have been made to classify acromion’s morphologic appearance on standard radiographs. While Bigliani et al. [1] (acromion type) and Aoki et al. [2] (acromial tilt) described the anatomical shape of the acromion, the lateral extension including the lateral acromial angle (LAA) [3], the acromion index (AI) [4], and the critical shoulder angle (CSA) [5] have been reported in more recent studies.

Higher incidences of rotator cuff tears (RCT) were reported by Bigliani et al. [1] with hooked (type-III) compared to curved (type-II) or flat (type-I) acromions. Furthermore, patients with RCT showed lower LAA [3] and a wide lateral extension of the acromion [4]. A flatter anterior slope of the acromion as described by Aoki et al. [2] was found in patients with impingement (IM) syndrome compared to asymptomatic individuals; while no significant association was detectable between a lower AI and patients with glenohumeral osteoarthritis (OA) [4]. The CSA, the most recent technique, which combines the measurement of glenoid inclination and lateral extension of the acromion, has been reported to indicate a greater value in patients with RCT compared to OA as well as to controls [5]. Certain radiological measurable characteristics of the acromial morphology seem to be associated with certain shoulder pathologies [5, 6]. Although, pathomechanisms of shoulder diseases are still not fully understood, biomechanical research demonstrated that during shoulder abduction, glenoid compression and joint shear forces depend on CSA, thus might explain different stress and wear patterns of the shoulder joint [7]. To our knowledge there is no study comparing different measurement methods in five different shoulder pathologies assessing the potential influence of acromion morphology.

The aim of the present study was two-fold: (1) to determine the most reliable radiological measurement technique (CSA, AI, or LAA) to describe acromion morphology in five different shoulder pathologies (CTA, OA, RCT, IM and tendinitis calcarea, TC) and (2) to evaluate the pathology-specific predictive values of the most reliable radiological parameter in combination with other potentially relevant factors.

## Methods

A total of 1000 patients from two hospitals including 500 patients (derivation cohort), who underwent surgery at our institution between 2005 and 2014, and 500 patients (external validation cohort), who underwent surgery between 2007 and 2014 at another hospital (Paracelsus Medical University, Salzburg, Austria) with a preoperative true anteroposterior radiograph showing the humerus in neutral position or up to 20° internal rotation were included in this retrospective study.

The groups consisted of patients with CTA Hamada stage 3 to 5, primary glenohumeral OA, posterosuperior RCT with an acromiohumeral interval of >7 mm and a chronic full-thickness tear of at least the supraspinatus tendon, IM with an acromial spur, and TC with an obvious calcium deposit in the subacromial space. All patients with CTA were treated with reverse total shoulder arthroplasty. Patients with glenohumeral OA underwent anatomical total shoulder arthroplasty and had an intact rotator cuff as examined during surgery. Arthroscopic rotator cuff repair was performed in RCT. The acromial spur was removed for patients with IM. Patients with TC were treated by arthroscopic removal of the calcium deposit. Exclusion criteria implied history of trauma, osteonecrosis, rheumatic, and other inflammatory diseases. Patients with bone neoplasm, collapse of the humeral head, severe glenoid erosion, and patients, who underwent previous surgery of the shoulder joint on the affected side, were also not included for analysis. Instead of healthy controls we included patients suffering from TC. Firstly to avoid unnecessary radiation of healthy subjects, secondly because of the existing preoperative radiographs as well as surgically controlled intact rotator cuff, and thirdly because TC is not described as a pathogenetic factor to further develop to CTA, OA, RCT [8], or IM.

In a first step, analyses of the derivation cohort were performed to determine the most appropriate radiological measurement to assess acromion morphology and to develop the prediction model by using epidemiological factors (gender, involved shoulder side, age, and body mass index) and radiological data. In the next step, the predication model was applied to the external validation cohort. The study was approved by the institutional review board (201407_EK06). A waiver for the informed consent was granted due to the fact that this was a retrospective study of data, which were already collected for clinical purposes.

### Radiographic assessment

All patients underwent preoperative standardized true-anteroposterior radiographs, which were digitally available. The medical imaging program Syngo.via (Siemens, Erlangen, Germany) for the derivation cohort and Impax EE R20 VIII (Agfa HealthCare, Mortsel, Belgium) for the external validation cohort were used to assess digital angle and distance measurements. According to Nyffeler et al. [4] the humeral head was required to be in neutral position or up to max. 20° internal rotation for being included into the definition of true a.p. radiograph. Supplementary scapula position must not exceeded 20° of either external or internal rotation [5]. Deviations smaller than these threshold values were confirmed as irrelevant in previous studies [4, 5].

**Fig. 1:**
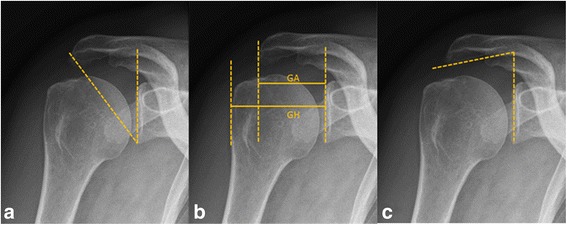
True-anteroposterior radiographs of a patient diagnosed with rotator cuff tear (RCT) of the *right shoulder*. **a** The critical shoulder angle (CSA of 38.8°), which was measured by drawing one line connecting the superior and inferior osseous margins of the glenoid cavity and then another line from the inferolateral border of the acromion and intersected the *first line *at the inferior glenoid margin. The angle between the *two lines* results in the CSA. **b** The acromion index (AI of 0.72) was calculated by dividing the distance of the glenoid plane to the lateral acromion border (GA) by the distance of the glenoid plane to the lateral margin of the humeral head (GH). **c** The lateral acromion angle (LAA of 76.05°) was assessed at the intersection of *two lines* representing the glenoid cavity and the acromion’s undersurface

Three different measurement techniques including the critical shoulder angle (CSA, Fig. 1a), acromion index (AI, Fig. 1b), and lateral acromion angle (LAA, Fig. 1c) were used to determine the acromial morphology in the coronal plane.

#### Statistical analysis

Patients’ characteristics are presented using descriptive statistics. We applied the Kolmogorov-Smirnov test to test continuous variables for normality. Normally distributed continuous variables were expressed as mean ± standard deviation; non-normally distributed continuous variables as median and range; and categorical data were presented using absolute frequencies and percentages. The chi-square or Fisher exact test was used for discrete variables (e.g. gender, shoulder side) and the independent *t*-test was used to explore differences between shoulder pathologies and continuous parameters (e.g. age, body mass index, CSA, AI, and LAA). The reproducibility of CSA measurements was examined in a subgroup of 100 (20 per shoulder pathology) randomly selected and blinded radiographs using intraclass correlation coefficients (ICCs) regarding inter- (two observers) and intra-rater (two measurements at different time points by one observer) reliability.

In order to calculate correlation between CSA, AI, and LAA the Person correlation coefficient (PCC) was used. The area under the receiver operating characteristic curve (AUROC) was obtained to evaluate the ability of CSA, AI, and LAA to distinguish between two shoulder pathologies. LAA was transformed by multiplication with −1 to fit into the AUROC. For further analyses, only significant different parameters between two shoulder pathologies were included.

Multinomial logistic regression was used to further evaluate the association between shoulder pathologies (dependent variable with five categories) and predictors (independent variables) including (a) the radiological measurement with the highest AUROCs (CSA, AI, or LAA) and (b) those parameters (gender, shoulder side, age, or body mass index), which were significantly associated with shoulder pathologies. TC was used as the reference category, since it has not been described as a pathogenic factor of the other diagnosis-related groups [8]. Model’s performance was statistically evaluated. The prediction model was derived from the development cohort and applied to the external validation cohort.

For all patients, sensitivity (e.g. proportion of patients with CTA classified by the model as having CTA) and specificity (e.g. proportion of patients without CTA classified by the model as not having CTA) were estimated to assess the model’s performance.

Data was analyzed using statistics software (SPSS version 21, IBM, New York, USA). Computed *p* values were two-sided and statistical significance was set at *p* < 0.05.

## Results

Patients’ characteristics of the development and external validation cohorts stratified by shoulder pathologies are shown in Table 1. Baseline characteristics were similar between both cohorts with some exceptions: Patients with RCT in the development cohort were significantly older (*p* < 0.001) and had less left shoulders involved (*p* = 0.013) than in the external validation cohort; and there were significantly more females than males in the development cohort (CTA: *p* < 0.001; TC: *p* = 0.008; TOTAL: *p* = 0.001).

**Table 1:** Patients characteristics

Cohort	Shoulder pathology	N	Age (years)	Gender (female/male)	Shoulder side (right/left)
Development	CTA	100	75 ± 8	75/25	70/30
	OA	100	69 ± 10	67/33	40/60
	RCT	100	65 ± 7	48/52	62/38
	IM	100	53 ± 11	44/56	55/45
	TC	100	47 ± 8	73/27	49/51
	TOTAL	500	62 ± 14	307/193	276/224
External Validation	CTA	100	77 ± 9	47/53	67/33
	OA	100	71 ± 10	57/43	45/55
	RCT	100	60 ± 9	44/56	79/21
	IM	100	51 ± 9	50/50	54/46
	TC	100	46 ± 7	54/46	56/44
	TOTAL	500	61 ± 15	252/248	301/199

Abbreviations: CTA, cuff tear arthropathy; IM, impingement; OA, osteoarthritis; RCT, rotator cuff tear; TC, tendinitis calcarea

A comparison of radiological measurements of 100 patients per shoulder pathology revealed that the majority of radiological parameters were significantly different between shoulder pathologies (Table 2) and all measurements correlated with each other (CSA and AI: PCC = 0.746, *p* < 0.001; CSA and LAA: PCC = −0.661, *p* < 0.001; AI and LAA: PCC = −0.495, *p* < 0.001). No differences with all three radiological parameters were detected between CTA and IM. Similar CSA, AI, and LAA were detected between RCT and IM, OA and TC, IM and TC, respectively (Table 2).

**Table 2:** Comparison of three radiological parameters to measure acromion morphology between five shoulder pathologies

	Critical Shoulder Angle (CSA)	Acromion Index (AI)	Lateral Acromion Angle (LAA)
**Cuff tear arthropathy** (CTA, n = 100)	35.2° ± 2.8°	0.69 ± 0.07	82.0° ± 6.3°
**Osteoarthritis** (OA, n = 100)	27.3° ± 3.5°	0.63 ± 0.09	89.5° ± 5.9°
**Rotator cuff tears** (RCT, n = 100)	36.3° ± 2.7°	0.74 ± 0.06	76.7° ± 5.8°
**Impingement** (IM, n = 100)	35.9° ± 2.7°	0.70 ± 0.05	83.2° ± 6.6°
**Tendinitis calcarea** (TC, n = 100)	30.2 ± 2.9	0.63 ± 0.06	84.2° ± 5.3°
*CTA vs. OA*	***p*** **< 0.001**	***p*** **< 0.001**	***p*** **< 0.001**
*CTA vs. RCT*	***p*** **= 0.006**	***p*** **< 0.001**	***p*** **< 0.001**
*CTA vs. IM*	*p* = 0.085	*p* = 0.689	*p* = 0.225
*CTA vs. TC*	***p*** **< 0.001**	***p*** **< 0.001**	***p*** **= 0.011**
*OA vs. RCT*	***p*** **< 0.001**	***p*** **< 0.001**	***p*** **< 0.001**
*OA vs. IM*	***p*** **< 0.001**	***p*** **< 0.001**	***p*** **< 0.001**
*OA vs. TC*	***p*** **< 0.001**	*p* = 0.842	***p*** **< 0.001**
*RCT vs. IM*	*p* = 0.289	***p*** **= 0.001**	***p*** **< 0.001**
*RCT vs. TC*	***p*** **< 0.001**	***p*** **< 0.001**	***p*** **< 0.001**
*IM vs. TC*	***p*** **< 0.001**	***p*** **< 0.001**	*p* = 0.235

*P* Values < 0.05 are displayed in bold

According to AUROC analyses, CSA was an overall more accurate parameter to distinguish between the different shoulder pathologies than AI, and LAA (Table 3). We therefore decided to include CSA instead of AI or LAA for further analyses.

**Table 3:** Area under receiver operating curve of three different radiological acromion morphology measurements between diagnosis-related groups

	Critical shoulder angle (CSA)		Acromion index (AI)		Lateral acromion angle (LAA)	
	AUROC^a^ (95% CI)	*P*	AUROC^a^ (95% CI)	*P*	AUROC^a^ (95% CI)	*P*
CTA vs. OA	**0.97 (0.95–0.99)**	<0.001	0.72 (0.64–0.79)	<0.001	0.81 (0.75–0.87)	<0.001
CTA vs. RCT	0.61 (0.54–0.69)	0.005	0.71 (0.63–0.78)	<0.001	**0.74 (0.67–0.80)**	<0.001
CTA vs. TC	**0.89 (0.84–0.93)**	<0.001	0.75 (0.69–0.82)	<0.001	0.60 (0.52–0.68)	0.013
CTA vs. IM	**0.58 (0.50–0.66)**	0.059	0.51 (0.69–0.82)	0.768	0.44 (0.36–0.52)	0.169
OA vs. RCT	**0.99 (0.98–0.99)**	<0.001	0.84 (0.79–0.90)	<0.001	0.93 (0.90–0.97)	<0.001
OA vs. TC	0.73 (0.66–0.80)	<0.001	0.51 (0.42–0.59)	0.908	**0.76 (0.69–0.82)**	<0.001
OA vs. IM	**0.98 (0.97–0.99)**	<0.001	0.75 (0.67–0.82)	<0.001	0.77 (0.70–0.83)	<0.001
RCT vs. TC	**0.93 (0.90–0.97)**	<0.001	0.91 (0.87–0.94)	<0.001	0.83 (0.77–0.88)	<0.001
RCT vs. IM	0.55 (0.47–0.63)	0.224	0.74 (0.67–0.82)	<0.001	**0.78 (0.71–0.84)**	<0.001
TC vs. IM	**0.93 (0.89–0.96)**	<0.001	0.80 (0.74−0.86)	<0.001	0.54 (0.46−0.62)	0.329

Abbreviations: CI, confidence interval; CTA, cuff tear arthropathy; IM, impingement; OA, osteoarthritis; RCT, rotator cuff tear; TC, tendinitis calcarea

^a^The highest AUROC are outlined in bold

Additional analysis revealed that intra- and inter-rater reliability for CSA measurements was excellent (Table 4).

**Table 4:** Intra- and inter-rater reliability for critical shoulder angle measurements

	ICC_intra_	ICC_inter_
Cuff tear arthropathy (CTA, *n* = 20)	0.913	0.943
Osteoarthritis (OA, *n* = 20)	0.983	0.915
Rotator cuff tears (RCT, *n* = 20)	0.990	0.971
Impingement (IM, *n* = 20)	0.996	0.987
Tendinitis calcarea (TC, *n* = 20)	0.975	0.942
TOTAL (*n* = 100)	0.987	0.982

From all potentially possible explanatory parameters (other than CSA), only age was detected to be significant different between the five shoulder pathologies compared to each other (Table 5).

**Table 5:** Patient characteristics potentially associated with five shoulder pathologies

Variable	Cuff tear arthropathy (CTA, *n* = 100)	Osteo- arthritis (OA, *n* = 100)	Rotator cuff tears (RCT, *n* = 100)	Impingement (IM, *n* = 100)	Tendinitis calcarea (TC, *n* = 100)	Comparison between groups
Gender (female/male)	75/25	67/33	48/52	44/56	73/27	*p* < 0.001^§^
CTA vs. OA/RCT/IM/TC		*p* = 0.275^†^	*p* < 0.001^†^	*p* < 0.001^†^	*p* = 0.872^†^	
OA vs. RCT/IM/TC			*p* = 0.010^†^	*p* = 0.002^†^	*p* = 0.441^†^	
RCT vs. IM/TC				*p* = 0.670^†^	*p* < 0.001^†^	
IM vs. TC					*p* < 0.001^†^	
Shoulder side (right/left)	70/30	40/60	62/38	55/45	49/51	*p* < 0.001^§^
CTA vs. OA/RCT/IM/TC		*p* < 0.001^†^	*p* = 0.296^†^	*p* = 0.041^†^	*p* = 0.004^†^	
OA vs. RCT/IM/TC			*p* = 0.003^†^	*p* = 0.047^†^	*p* = 0.255^†^	
RCT vs. IM/TC				*p* = 0.389^†^	*p* = 0.087^†^	
IM vs. TC					*p* = 0.479^†^	
Age (years)	74.7 ± 7.5	69.3 ± 9.7	64.6 ± 7.4	53.0 ± 10.7	47.1 ± 8.4	*p* < 0.001^§^
	75 (55–89)	68 (44–91)	64 (48–80)	52.5 (29–79)	47 (30–66)	
CTA vs. OA/RCT/IM/TC		*p* < 0.001^‡^	*p* < 0.001^‡^	*p* < 0.001^‡^	*p* < 0.001^‡^	
OA vs. RCT/IM/TC			*p* < 0.001^‡^	*p* < 0.001^‡^	*p* < 0.001^‡^	
RCT vs. IM/TC				*p* < 0.001^‡^	*p* < 0.001^‡^	
IM vs. TC					*p* < 0.001^‡^	
BMI (kg/m^2^)	28.4 ± 4.7	29.9 ± 5.1	27.9 ± 4.4	26.8 ± 4.9	25.2 ± 4.1	*p* < 0.001^§^
	27.6 (19–43)	29.7 (20–48)	27.5 (19–41)	26.0 (18–42)	24.8 (18–43)	
CTA vs. OA/RCT/IM/TC		*p* = 0.035^‡^	*p* = 0.410^‡^	*p* = 0.014^‡^	*p* < 0.001^‡^	
OA vs. RCT/IM/TC			*p* = 0.003^‡^	*p* < 0.001^‡^	*p* < 0.001^‡^	
RCT vs. IM/TC				*p* = 0.081^‡^	*p* < 0.001^‡^	
IM vs. TC					*p* = 0.018^‡^	

^†^ *P* values were calculated with Chi^2^-test or Fisher exact t-test. ^‡^ *P* values were calculated with the independent *t*-test. ^§^ *P* values were calculated using ANOVA

The multinomial logistic regression model using CSA and age as predictors for shoulder pathologies and TC as reference demonstrated that the odds of OA versus TC decreased by 13.4% for every 1° CSA decrease, while the odds of CTA, RCT, and IM versus TC significantly increased more than twice (Table 6).

**Table 6:** Multinomial logistic regression model^b^ for the association of critical-shoulder-angle and age of major shoulder pathologies

		OR (95% CI)^a^	*P* value
Cuff tear arthropathy (CTA)	CSA	2.62 (2.07 to 3.31)	<0.001
	age	1.52 (1.42 to 1.63)	<0.001
Glenohumeral osteoarthritis (OA)	CSA	0.87 (0.75 to 1.01)	0.060
	age	1.26 (1.19 to 1.34)	<0.001
Rotator cuff tear (RCT)	CSA	2.77 (2.22 to 3.47)	<0.001
	age	1.30 (1.23 to 1.38)	<0.001
Impingement (IM)	CSA	2.38 (1.93 to 2.93)	<0.001
	age	1.12 (1.07 to 1.18)	<0.001

Abbreviations: OR, odds ratio; CI, confidence interval; CSA, critical shoulder angle

^a^The reference category was tendinitis calcarea (TC)

^b^The model yielded in the overall test of relationship (model fitting to raw data) an excellent Chi^2^ of 851.8 (degrees of freedom = 8, *p* < 0.001), an overall classification accuracy of 64.8%, and a strength (Pseudo R^2^) according to Cox und Snell of 0.818, Nagelkerke of 0.852, McFadden of 0.529

**Fig. 2:**
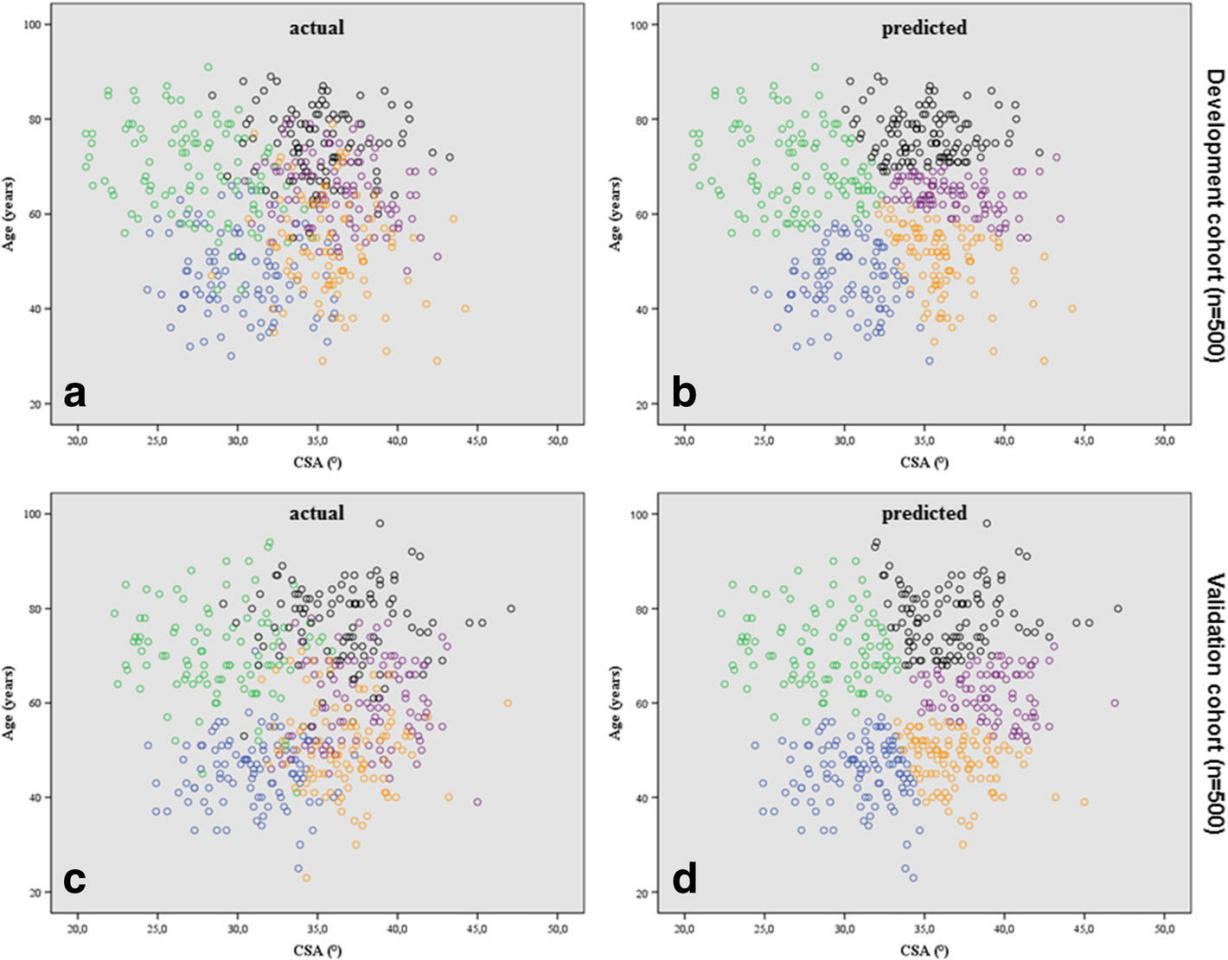
Scatterplots showing critical shoulder angles (CSA). CSA and age grouped according to actual (**a**, **c**) and predicted (**b**, **d**) shoulder pathologies including cuff tear arthropathy (CTA, *black circles*), osteoarthritis (OA, *green circles*), rotator cuff tear (RCT, *purple circles*), impingement (IM, *orange circles*), and tendinitis calcarea (TC, *blue circles*) of the development (**a**, **b**) and external validation (**c**, **d**) cohort

The spread of all measured CSA plotted against age, grouped into actual and predicted shoulder pathologies of the development and validation cohort, are represented in Fig. 2.

Both, the development as well as the validation cohort distribution of actual CSA and age values showed a similar CSA-age pattern (Fig. 2a and c). Generally, two age dependent pathways were recognized: Firstly, patients with higher CSA (above approximately 30°) and increasing age showed a pathology pattern from IM to RCT to CTA. The second pattern (CSA below approximately 30°) included patients with presumably “normal” acromion morphology (i.e. non-rotator cuff compromising acromion morphology as in TC) showed a development of OA with increased age.

The model correctly predicted shoulder pathologies in 64.8% for the development cohort and in 70.6% for the validation cohort. The overall model’s performance to predict five shoulder pathologies of 1000 patients was 67.7%. Further details regarding sensitivity and specificity of the shoulder pathologies are provided in Table 7. While the model’s performance was fairly high regarding specificities (all above 70%), not all sensitivities for the five shoulder pathologies were above 70%.

**Table 7:** The model’s performance to predict five shoulder pathologies

OBSERVED	PREDICTED	Cuff tear arthropathy (CTA)	Osteoarthritis (OA)	Rotator cuff tears (RCT)	Impingement (IM)	Tendinitis calcarea (TC)
Cuff tear arthropathy (CTA)		71.5% (*n* = 143)	7.5% (*n* = 15)	18.5% (*n* = 37)	2.0% (*n* = 4)	0.5% (*n* = 1)
Osteoarthritis (OA)		7.5% (*n* = 15)	82.0% (*n* = 164)	0.5% (*n* = 1)	2.0% (*n* = 4)	8.0% (*n* = 16)
Rotator cuff tears (RCT)		22.5% (*n* = 45)	2.5% (*n* = 5)	47.5% (*n* = 95)	24.0% (*n* = 48)	3.5% (*n* = 7)
Impingement (IM)		6.0% (*n* = 12)	2.0% (*n* = 4)	23.0% (*n* = 46)	58.0% (*n* = 116)	11.0% (*n* = 22)
Tendinitis calcarea (TC)		0.0% (*n* = 0)	7.5% (*n* = 15)	0.0% (*n* = 0)	13.0% (*n* = 26)	79.5% (*n* = 159)
*Sensitivity* ^*a*^		*71.5%*	*82.0%*	*47.5%*	*58.0%*	*79.5%*
*Specificity* ^b^		*72.8%*	*76.1%*	*71.6%*	*71.8%*	*75.4%*

^a^Sensitivity correctly categorized (observed = predicted); ^b^Specificity correctly categorized (not observed = not predicted)

## Discussion

The present study demonstrates that CSA and age had the most predictive capabilities for CTA, OA, RCT, IM, and TC. We supported previous findings regarding the excellent sensitivity and specificity of CSA and the superiority compared to AI and LAA. Furthermore, a typical CSA-age pattern was detected in 1000 patients from the two orthopedic clinics. The developed prediction model using CSA and age as predictors was able to correctly predict shoulder pathologies in 70.6% of the validation cohort.

**Fig. 3:**
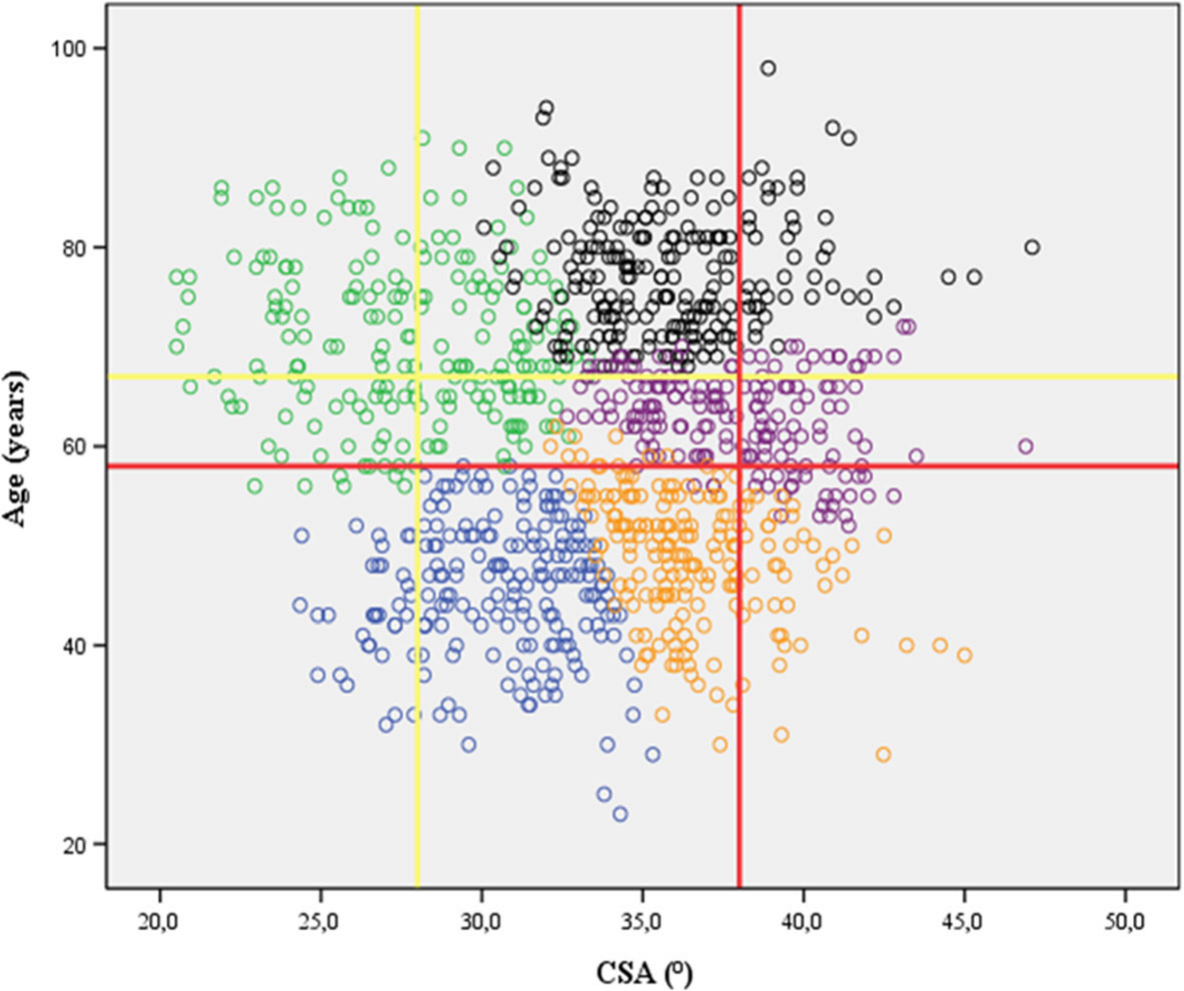
Scatterplots showing critical shoulder angles (CSA). CSA and age grouped according to predicted shoulder pathologies including cuff tear arthropathy (CTA, *black circles*), osteoarthritis (OA, *green circles*), rotator cuff tear (RCT, *purple circles*), impingement (IM, *orange circles*), and tendinitis calcarea (TC, *blue circles*) of both the development and the external validation cohort. The *red lines* mark a 58 year old patient with a CSA of 38° and the *yellow lines* mark a 67 year old patient with a CSA of 28°

Acromion morphology, as already previously reported, is a powerful predictor for RCT and OA [5, 6]. We confirm the results of Moor et al. [5, 9] that patients with rotator cuff disease have a significant greater CSA than patients with primary OA. Mean CSA of primary OA patients showed similar results in both studies (27° versus 28° [6]). The CSA for patients with RCT was differing by 2° compared to Moor et al. [5, 9] (38° versus our 36°). According to our prediction model (predicted shoulder pathologies of all 1000 patients are given in Fig. 3), mean CSA and age reported by Moor et al. [5] would correctly predict OA for a 67-year old patient with a CSA of 28° (yellow line Fig. 3); however for a 58-year old patient with a CSA of 38° it would predict either RCT or IM (red line Fig. 3). This discrepancy may be explained by the similar pathogenetic mechanism of rotator cuff tear formation.

Additionally, our results revealed that CTA and IM can also be associated with a certain CSA. However, but not surprisingly, CSA was not significantly different between CTA vs. IM and RCT vs. IM. It is believed that mechanical IM further develops over the years to RCT and RCT may further develop to CTA [10]. Hence, patients’ age contributed to a better distinction between the three shoulder pathologies. Results of the present study might further contribute to the theory of the rotator cuff pathology cascade. Moreover according to this model, patients from the control group might develop OA with increasing age.

Furthermore, it must be questioned if shortening the lateral acromion (decreasing the CSA) by surgical intervention is able to reduce the development of certain shoulder pathologies. A recent study by Garcia et al. [11] showed that the risk of retear after rotator cuff repair increased significantly with a CSA >38°.

Previous biomechanical findings showed a more superiorly directed deltoid force vector in patients with wide lateral acromion and a concomitant increased requirement of supraspinatus force to stabilize the humeral head [4, 8, 12]; whereas a shorter acromion extension produced higher glenohumeral joint reaction forces potentially leading to OA [13]. A wider lateral extended acromion was expected in CTA accompanying with superior migration of the humeral head. In contrary to our assumptions, CTA patients had a significantly smaller lateral extension than RCT patients and showed an equal CSA as asymptomatic controls of previous studies [5, 14, 15]. In our study the main pathomechanism of CTA could not only be explained by acromion morphology. Geometrically, the CSA is also influenced by superior glenoid inclination, which is especially present in CTA. The more the glenoid superiorly inclines, the higher the CSA. This fact should lead to a higher CSA for CTA than actually detected in our study. We assume that subscapularis failure is a complemental factor leading to superior humeral head migration and subsequently to CTA. Superior glenoid inclination seems to develop as a consequence of long standing superior migration due to the rotator cuff rupture.

In contrast to a similar study [5, 6] investigating CSA using patients with orthopedic problems other than the shoulder, we included patients with TC – assuming that the origin of the calcium formation in any presumed mechanism of pathogenesis is without any association to the acromion morphology [16] - as our control group. Interestingly, the CSA of our controls was lower (Ø 30°) than those in the study by Moor et al. (Ø 33°). We assume that the differences could be explained by the fact that our controls were nearly 20 years younger (Ø 47 versus 66 years). It could be speculated that based on such findings acromion morphology is changing over time. Thus, taking younger patients as controls might be an advantage as - apart from the biomechanical influence of the acromion morphology - other degenerative processes such as degenerative cartilage and tendon diseases, which contribute to the origin of shoulder pathologies, are less frequent.

The overall predictive performance of the model provided a reasonable accuracy to correctly predict shoulder pathologies. Interestingly, the prediction model was able to better predict shoulder pathologies in the validation (71%) than in the development cohort (65%). However, while it performs well in correctly identifying CTA, OA, and TC, it has difficulties to classify IM (sensitivity: 58%) and even more RCT (sensitivity: 48%). Such facts might be attributed to the combination of extrinsic and intrinsic factors leading to progressive tendon degeneration. IM is known to be caused not only by the acromial shape, but also by altered scapulathoracal motion [17]. The low sensitivity of the prediction model regarding RCT can be explained by the gross definition of RCT in our study, which is in contrast to a recent study reporting that the CSA combined with age and trauma can precisely predict the integrity of the posterosuperior rotator cuff [6]. Although Moor et al. [6] included only supraspinatus integrity in their study, no intraoperative confirmation was described. Because of the complex anatomy of the rotator cuff’s footprint accurate description of tear configuration solely from magnetic resonance imaging (MRI) is difficult. As the development of most shoulder pathologies is multifactorial, for a more accurate prediction model more accurate pathology-specific factors as well as precisely defined shoulder pathologies should be considered in future studies. Still, the advantage of the present study is that all pathologies were confirmed intraoperatively.

The study exhibits several limitations. We have to consider a selection bias because only symptomatic patients were included, thus potentially limiting the usefulness of this prediction model for a wider population such as healthy asymptotic controls or patients with other shoulder pathologies. Furthermore, the RCT group was not divided into postero-superior and antero-superior tears, which may influence the model’s accuracy; yet in a primary care setting it is rather difficult for non-specialists as well as often also for specialists and especially without MRI to distinguish between different RCT patterns.

Despite our promising findings in this large study, we are aware that our prediction model is not 100% accurate and further research using prospectively collected data including more potential predictors as well as a more detailed pathology definition (i.e. postero-superior vs. antero-superior RCT,…) and wider ranges of shoulder diseases is essential.

## Conclusion

In summary, the present study showed that CSA and age, two easily assessable variables, adequately predict shoulder pathologies in patients with shoulder complaints. Using the prediction model in primary care may be considered since severe shoulder pathologies might be detected earlier, thus early adequate treatment by a specialist could be applied.

## Abbreviations

AI: Acromion index
AUROC: Area under the receiver operating characteristic curve
CSA: Critical shoulder angle
CTA: Cuff tear arthropathy
ICC: Intraclass correlation coefficient
IM: Impingement
LAA: Lateral acromial angle
MRI: Magnetic resonance imaging
OA: Osteoarthritis
PCC: Person correlation coefficient
RCT: Rotator cuff tear
TC: Tendinitis calcarea
